# Pattern of animal bites and post exposure prophylaxis in rabies: A five year study in a tertiary care unit in Sri Lanka

**DOI:** 10.1186/s12879-016-1394-5

**Published:** 2016-02-04

**Authors:** Senanayake Abeysinghe Mudiyanselage Kularatne, Dissanayake Mudiyanselage Priyantha Udaya Kumara Ralapanawa, Koasala Weerakoon, Usha Kumari Bokalamulla, Nanada Abagaspitiya

**Affiliations:** 1 Department of Medicine, University of Peradeniiya, Peradeniya, Sri Lanka; 2 Department of Parasitology, Rajarata University, Mihintale, Sri Lanka; 3 Teaching Hospitals, Peradeniya-Kandy, Sri Lanka

**Keywords:** Rabies, Post exposure treatment, Rabies vaccine, Rabies immunoglobulin, Sri Lanka

## Abstract

**Background:**

Rabies is a global problem which occurs in more than 150 countries and territories including Sri Lanka, where human deaths from rabies are in decline whilst resources incurred for prevention of rabies are in sharp incline over the years. In this backdrop, we aim to audit the post-exposure treatment (PET) in rabies and the pattern of animal bites in a tertiary care hospital in Sri Lanka.

**Methods:**

This study was carried out at Teaching Hospital Peradeniya (THP), in the Central Province of Sri Lanka from 2007-2012 where a registry of all PET has been maintained. The data from registries were extracted after obtaining permission from the hospital authority for analysis.

**Results:**

There were 19 661 cases of animal exposure presented to the THP over the study period of 5 years. Of them, the majority-17431(88.66 %) were definitive animal bites whilst scratches accounted for 2147(10.92 %) and 83(0.42 %) were miscellaneous exposures. According to the severity grading of injuries, 7 362(37 %) were major bites and 12 226(62 %) were minor bites. The domestic unvaccinated dogs and cats were responsible for 10,662 (54 %) and 3,982 (20 %) of exposures respectively. The total cost incurred for both anti-rabies vaccine and anti rabies serum during the study period is 24,795,888.00 Sri Lankan rupees (190,737.60US$).

**Conclusions:**

The pattern of animal bite shows high dominance of domestic dogs and cats exposures. The age of victims ranged from infancy to old-age with higher incidence among children. Even though PET is costly, continued surveillance and rabies control is still necessary along with public education and vaccination of domestic pets.

## Background

Rabies is a global problem which occurs in more than 150 countries and territories [1, 2]. It is a vaccine preventable viral zoonotic infection caused by lyssavirus of the Rhabdoviridae family, currently responsible for tens of thousands of deaths every year, mostly in Asia and Africa [1, 2]. The infection prevails among domestic and wild animals, of them dogs are the main source of the human rabies transmitted by close contact with infectious material, usually saliva, bites or scratches [1].

Sri Lanka is a tropical island situated in the Indian Ocean with rich diversity of flora and fauna. The current population is about 20.2 million, of this 18.3 % living in urban areas, 77.3 % in rural areas and 4.4 % living in the estate areas [3]. Sri Lanka is one Asian country where human deaths from rabies has decreased markedly during the past decade, however, rabies is still endemic and remains a significant public health problem [3]. The Ministry of Health of Sri Lanka spends substantial amount of its health budget on anti-rabies treatment in humans. Recent estimates are that the cost of post-exposure prophylaxis (PEP) per patient is 173 and 177 US dollars without immunoglobulin or with immunoglobulin respectively [3, 4].

All mammals are susceptible to rabies, but some animal species do not serve as a reservoir species for rabies virus and therefore do not normally play a role in transmission of the virus [3]. The most important reservoir animal for maintenance of rabies virus and transmission to humans is the domestic dog, with over 90 % of human cases attributable to dog bites [4]. Dogs are the source of infection in all human rabies deaths in Asia and Africa whilst bats are the source of most human rabies deaths in Americas [1]. Bat rabies has also recently emerged as a public health threat in Australia and western Europe [1]. Human deaths following exposure to foxes, raccoons, jackals, mongoose and other wild carnivore host species are very rare [1].

People are usually infected following a deep bite or scratch by an infected animal. Transmission can also occur when infectious material-usually saliva-comes into direct contact with human mucosa or fresh skin wounds. Human to human transmission by bite is theoretically possible but has never been confirmed [1]. Rarely, rabies may be contracted by inhalation of virus containing aerosol or via transplantation of an infected organs [1]. Ingestion of raw meat or other tissue from animals infected with rabies is not a source of human infection [1, 5].

Rabies is always fatal once symptoms develop. However, effective treatment soon after exposure to rabies can prevent the onset of disease. This is known as post exposure treatment(PET) [1, 6]. Recorded human deaths from rabies in the world has decreased significantly due to widespread vaccination of domestic dogs and cats and the development of human vaccines and immunoglobulin treatment [1, 6]. Post exposure treatment consist of local treatment of wound, a course of potent and effective rabies vaccine that meets World Health Organization(WHO) recommendations and the administration of rabies immunoglobulin if indicated (6,7,8 ). According to the guidelines issued by Ministry of Health, Sri Lanka, it is essential to screen the patient and the animal before the decision is made regarding PET. The exposure to animal can be categorized as major exposure and minor exposure. The major exposure is defines as single or multiple bites with bleeding in the head, neck, face. chest, upper arms, palms, tips of fingers, toes, genitalia, multiple deep scratches with bleeding in head, neck and face, single or multiple deep bites in any part of the body, contamination of mucus membranes with saliva and bites of wild animals with bleeding [7]. The minor exposure is defined as single superficial bite or scratch with bleeding in lower limbs, upper limbs, abdomen and neck, nibbling of uncovered skin, contamination of open wounds with saliva, single or multiple scratches without bleeding in any part of the body, drinking raw milk of rabid cow or goat [7]. After screening victim and animal and when decide to give PET all the patients in the major category should be given rabies immunoglobulin(equine or human) followed by a course of anti rabies vaccine(ARV). Patients in minor category should be given only a course of ARV [7].

No test is available to diagnose rabies infection in humans before the onset of clinical disease and unless the rabies specific signs of hydrophobia or aerophobia are present. The reference method for diagnosing rabies is the Fluorescent Antibody Test(FAT),which is recommended by the WHO and is used by Medical Research Institute(MRI) of Sri Lanka. The FAT relies on visualization of rabies antigen by florescent microscopy techniques. The diagnosis can be made by detecting the whole virus, viral antigens, viral specific antibodies in the cerebrospinal fluid or nucleic acid in infected tissues (brain, skin, urine or saliva). Rabies can be reliably diagnosed from brain samples taken after death. Cerebral inclusion bodies (Negri bodies) are 100 % diagnostic for rabies infection but are found in only about 80 % of cases [1, 3, 5, 8].

Sri Lanka is one of the fastest growing economies in Asia today and in recent years, she has experienced population growth, rapid urbanization, deforestation and construction of new highways, dams and irrigation systems [3]. All these changes can affect reservoir species habitat and may influence the epidemiology of rabies in different ways [3]. Our study was performed to identify the current pattern of animal bites and to evaluate PET at a tertiary care hospital in Sri Lanka.

## Methods

**Fig. 1:**
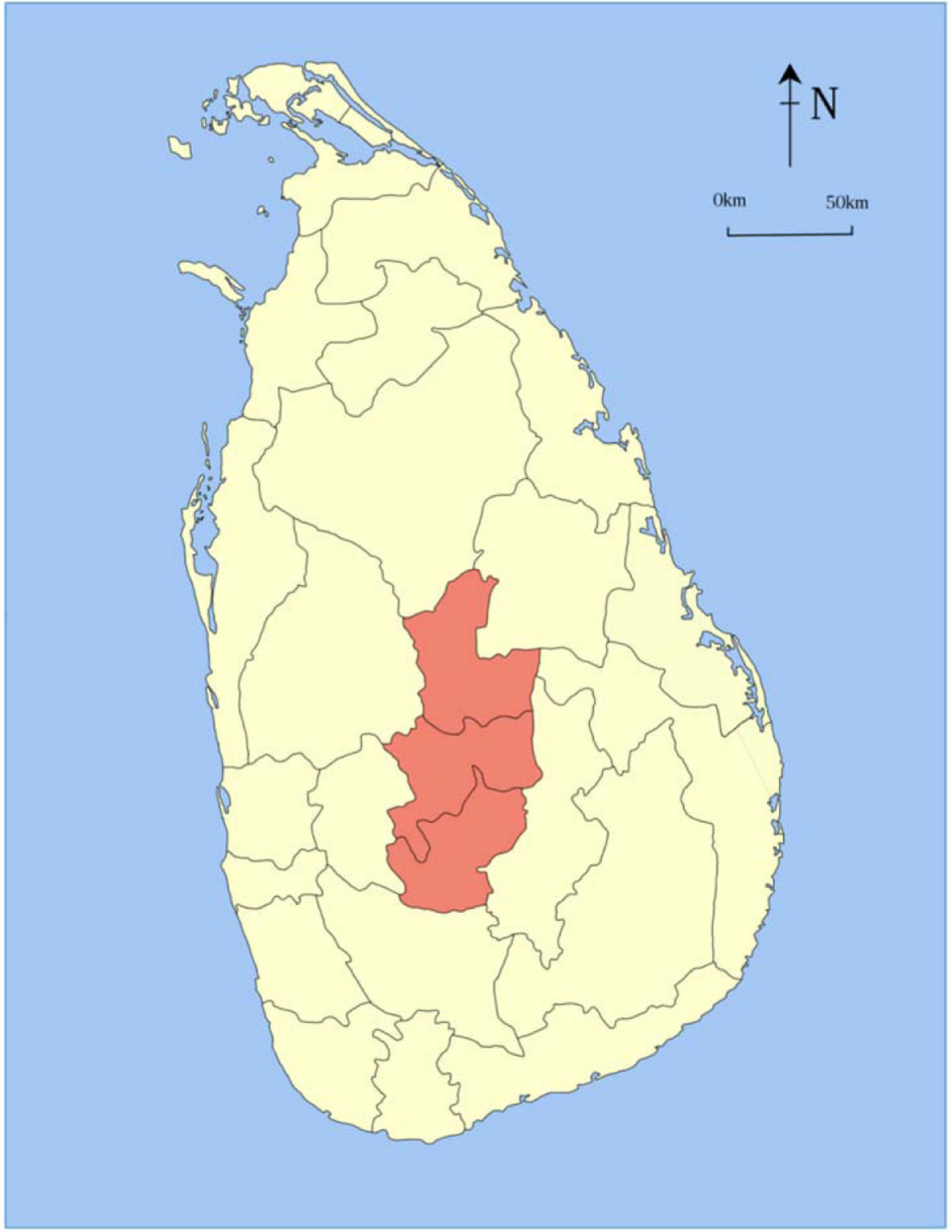
Central Province of Sri Lanka. Source: Home page –Central Province of Sri Lanka and copyright permission was obtained from the Provincial Director of Health Services, Central Province, Sri Lanka

This is an observational/descriptive study carried out at Teaching Hospital Peradeniya (THP), in the Central Province Sri Lanka during the period of 2007-2008 and 2009-2012. The Central Province is located in the central hills of Sri Lanka (Fig. 1) and consists of the three districts-Kandy, Matale and Nuwara Eliya. It has 5,763 villages and local government bodies comprising 3 municipality councils and 6 urban council areas. In 2013 population was 2,558,716 and population density for Central Province was 459 persons per square kilometer. According to the census data 70.0 %, 20.2 % and 9.8 % 0f the population were classified as rural, estate and urban respectively.

The hospital has dedicated team of trained medical officers in the outpatient department to manage victims of animal exposure. A register had been maintained where basic details of all animal exposures were recorded that included residence, age, gender, location of bite wounds, care given to wound, animal involved and detail of PET. Post exposure treatment was started without delay according to the WHO criteria. Cold chain is well maintained. For the PET we have used two kinds of inactivated anti rabies cell culture vaccines namely Purified vero cell rabies vaccine(PVRV) and Purified chick embryo cell vaccine(PCEC). Intradermal(ID) inoculation of ARV was carried out according to the circular issued by Ministry of Health,Sri Lanka. Immunoglobulins used were both Equine rabies immunoglobulin (ERIG) and Human rabies immunoglobulin (HRIG) according to the guidelines issued by the Ministry of Health, Sri Lanka.Follow up data like outcome of the animal and PET management of victims were recorded. Animal information like species, their ownership, whether stray or feral, rabies vaccination status were recorded. In Sri Lanka dog immunization is carried out under the guidelines issued by Public Heath Vetenary Services of Ministry of Health.Vaccine is administered either subcutaneously or intramuscularly. Vaccination records should be kept securely with the owner and in case of animal bite owner should submit it to the hospital. We used these records to obtain the immunization status of dogs.

Data from registries were extracted after obtaining permission from the hospital authority. These data were entered into password protected computer and SPSS version 20 was used for statistical analysis.

## Results

There were 19 661 cases of animal exposure presented to the THP over the study period of 5 years with predominance of males and the children. (Table 1) The age of the cases ranged from 3 months to 96 years with the mean age of 29 years. Of them, 29.94 % were younger than 12 years and 10 819(55.02 %) were males.

**Table 1:** Distribution of injuries and type of animal within age categories

Description		Age categories							
		<12 years		13–20		21–60		>60	
		n	%	n	%	n	%	n	%
Total		5886		2340		9212		1683	
Gender	Male	3412	58	1311	56	4912	53	885	53
	Female	2474	42	1029	44	4300	47	798	47
Level of injury	Major	2090	36	862	37	3519	38	709	42
	Minor	3784	64	1477	63	5672	62	972	58
Type of injury	Bite	4878	83	2066	88	4831	52	1587	94
	Scratch	999	17	269	11	744	8	94	6
Type of animal	Dog	4078	69	1619	69	6709	73	1311	78
	Cat	1621	28	588	25	2092	23	319	19
	Other/Wild animals	182	3	131	6	368	4	48	3

According to the gender among the all age groups susceptibility of animal exposure more in male. (Male 55.02 %, Female 44.98 %). Of the type of animal exposure, the majority-17431(88.66 %) were definitive animal bites whilst scratches accounted for 2147(10.92 %) and 83(0.42 %) were miscellaneous exposures. According to the severity of injuries 7 362(37 %) were major bites and 12 226(62 %) were minor bites. (Table 1) All patients in major category were given rabies immunoglobulin (Equine or Human) followed by a course of Anti Rabies Vaccine. Equine rabies immunoglobulin was used in most of the patients. In cases where pre-administration skin sensitivity became positive, human rabies immunoglobulin has been given. Patient in the minor category were given a course of anti Rabies Vaccine only. For majority of the patients cell culture vaccine(PVRV) was used and when its stock is not available embryonated egg based vaccine (PCEC) was used for PET at THP during this study period. Nerve tissue vaccine was not used. Animal involved is summarized in Table 2. Accordingly domestic unvaccinated dogs and cats exposure was the main type of animal involvement. Animal involved were observed for 10 days and 15 503(79 %) were alive, 365(2 %) were dead and of 3742(19 %) outcome was not known.

**Table 2:** Categories of animal involved

Animal involved	No	Percentage(%)
Dogs-domestic; vaccinated	559	3
Dogs-domestic; unvaccinated	10 662	54
Dogs-stray	2 865	15
Cats-domestic vaccinated	15	0.1
Cats-domestic; unvaccinated	3 982	20
Cats-stray	724	4
Monkey	187	0.95
Squirrel	317	1.62
Bat	13	0.07
Bandicoot	100	0.5
Rabbit	17	0.09
Pig	24	0.12
Kalavedda/Pole cat	24	0.12
Mongoose	22	0.11
Pony	6	0.03
Goat,cattle,buffalo,wild animal	50	0.25

**Fig. 2:**
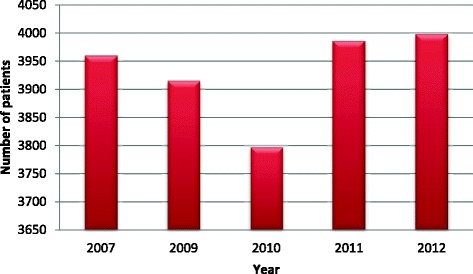
Annual distribution of the cases presented for PET at THP

**Fig. 3:**
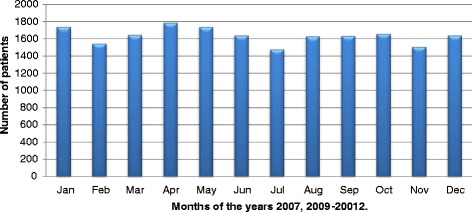
Cumulative monthly distribution of the cases presented for PET at THP during 2009 to 2012 and in 2007

Annual distribution of animal exposures over 5 year period shows increasing trend in 2011 and 2012 (Fig. 2). In 2010 there were 3800 cases presented for PET to THP. In 2011 there were 3975 and in 2012 there were 4000 cases presented for PET to THP. So there were 175 to 200 more cases presented for PET to THP in 2011 and 2012 respectively. This increase in number of cases presented for PET possibly due to increase in number of animal bites in the region, more awareness among public about importance of getting PET as a result of health education programs, due to increase number of dog population or due to increase number of animal exposure other than dogs. Cumulative monthly distribution of animal exposure/bite shows even pattern with more cases presenting in months of school holidays- April, August and December. The case load per month varies from 1 400 to 1 800 cases (Fig. 3)

.

WHO recommends 10-days observation of the animal after a suspected bite [9]. In our study 80.71 % of the animal were observed by their owner for 10 days after a suspected animal exposure, and 97.70 % of these were reported by the owners as ‘normal’ at the end of 10th day. From all(365) the dead animals brain samples were sent to the reference laboratory. Using both Seller stain for Negri bodies and fluorescent antibody test methods brain samples were evaluated and non were found to be rabies positive.

Of the 19 661 animal exposures, 19 521 cases (99.29 %) were included in a post-exposure rabies vaccination program. Among this 334(1.7 %) victims received less than 4 anti rabies vaccine (ARV) doses while 7030(35.76 %), 12 149(61.79 %) and 8(0.04 %) received 4, 6 and more than 6 ARV doses respectively. There were no major side effect reported following vaccine or immunoglobulin administration, about 11 % of victims developed minor reactions like transient erythema at sites of intradermal administration. Total expenditure for anti-rabies vaccine and anti-rabies serum during the study period is shown in Table 3. Accordingly total amount spent for anti-rabies vaccine and anti rabies serum during the study period is 24,795,888.00 Sri Lankan rupees (190,737.60US$).

**Table 3:** Total expenditure for antirabies vaccine and antirabies serum/immunoglobulin during the study period at Teaching Hospital, Peradeniya

Year	Cost for anti rabies vaccine (Sri Lankan rupees)	Cost for anti-rabies serum + Anti-rabies human immunoglobulin (Sri Lankan rupees)
2007	915 240.00	1 333 080.00
2009	1 904 640.00	1 314 565.00
2010	4 039 357.00	1 665 520.00
2011	3 377 241.00	6 056 270.00
2012	2 236 416.00	1 953 559.00
Total	12 472 894.00	12 322 994.00

## Discussion

We found that large numbers of animal bites have presented to a single tertiary care hospital in Sri Lanka for post exposure treatment against rabies and has incurred colossal cost. Extrapolation of these figures may help to judge approximate national burden in-terms of animal exposures and cost for PET. The neighboring main tertiary care hospital of the Province is General Hospital, Kandy which bears the burden 4 times more than the study centre-GH Peradeniya (unpublished data). (Table 4). These figures highlight the burden of animal exposure and post-exposure treatment in the Central Province, Sri Lanka.

**Table 4:** During the study period, number of cases presented to the Teaching Hospital, Kandy for PET for comparison

Year	No
2007	10597
2009	11683
2010	11735
2011	11969
2012	12305
Total	58289

The pattern of animal bite shows high dominance of dogs and cats followed by large gamut of other animals. Most of the domestic dogs and cats were not vaccinated against rabies that resulted high wastage of PET. Apparently, animal exposures were happening regularly over the years with high incidence during school vacations. The age range of the victims was far wide ranging from infancy to old-age, but the incidence was high among the children.

We aimed at describing animal exposure and post exposure prophylaxis over five years from 2007 to get a glimpse annual pattern. The data of 2008 was not included due to missing of a data register. During the study period number of human deaths due to rabies from the nine provinces of Sri Lanka is shown in Table 5 [10–14]. Accordingly human rabies cases from the Central Province ranged from 1–6 during the study period. None of these cases were reported from the THP. The decline of human rabies in the province could also be due to other strategies such as dog vaccination and their birth control programs.

**Table 5:** Number of human deaths due to rabies in different provinces in Sri Lanka

Year	2007	2008	2009	2010	2011	2012	2013
Province							
Central Province	6	3	3	2	1	2	0
Eastern Province	7	7	8	8	11	7	5
Northern	6	2	5	8	3	3	6
North Central Province	3	3	6	4	1	3	4
North Western Province	8	16	5	4	6	6	3
Sabaragamuwa Province	3	1	3	3	1	3	2
Southern Province	10	7	7	5	7	1	4
Uva Province	2	3	3	4	2	3	2
Western Province	14	9	18	11	9	10	2
Total	59	51	58	49	41	38	28

Source: Epidemiology Bulletin, Sri Lanka

The religious and cultural traditions in Sri Lanka promote compassion to animals and killing animals is considered a great sin. Even domestic dogs are set free to roam outside the houses where they socialize with stray dogs. Well-wishers and animal lovers feed dogs. Dumping garbage with leftover food in streets is a common practice in Sri Lanka that thrive all kinds of animals. These attitudes and practices have great hindrance to elimination of rabies from the land. This fact is testified by our finding that many bites were inflicted by dogs which roam every corners and streets of the villages and cities of the island. We found majority of victims exposed to animals were in young and middle age groups with predominance of males. This may probably be due to out-door exposure to dogs and other animals. According to the study of *Salve et al* majority of patients attending to anti-rabies clinic in Haryana were of younger age group and males [15]. A similar Study done by *Shah et al* reported 48.4 % of cases of animal bites were below 25 years [16]. We found dog and cats were responsible for 71 % and 24 % of animal bites. Similar findings had been reported by *Moore et al* where the incidence of dog bite was 75 % and 17.2 % for cats [17].

In situations where offending animals could be observed, we found 80 % animal were observed for 10 days after the bite and interestingly 98 % of them remained healthy at 10th day indirectly implying wastage of PET for healthy animal bites. However, in dreaded disease like rabies taking risk after animal bite is illogical despite unnecessary vaccination. However, by adhering to the national guideline of PET, discontinuation of vaccination had been practiced in cases of animals remains healthy beyond 10 days. In GH Peradeniya, we followed intradermal (ID) schedule of antirabies vaccine (ARV). Immunoglobulins used were both Equine rabies immunoglobulin (ERIG) and Human rabies immunoglobulin (HRIG) according to the guidelines issued by the Ministry of Health, Sri Lanka. Considering cost of PET in GH Peradeniya, cost for immunoglobulin was more than AVR. Of immunoglobulins, HRIG is more expensive and given due to fear of allergy and anaphylaxis of ERIG. Therefore, availability of less allergic ERIG is needed and measures should be developed to counter allergic reactions.

In Sri Lanka, 50–60 rabies deaths occur annually, mainly due to exposure to infected dogs. The estimated dog population density in the island is 108 dogs/km^2^, which is much higher than the threshold density of 4.5 dogs/km^2^ necessary for the persistence of rabies [3]. Rabies control measures launched in Sri Lanka since 1975 have had a tremendous effect on the incidence of human rabies. The number of human rabies deaths declined from 377 to 28 by 2013 [7]. Implemented strategies for rabies control in Sri Lanka include responsible dog/cat ownership, vaccination of all different groups of dogs, animal birth control, habitat control progrsmme, humane disposal of rabid and other susceptible animals and continuous monitoring and evaluation [7]. Mass campaigns for rabies vaccination of household dogs in Sri Lanka is carried out at puppies age of 6 weeeks,three month and then annually.If stray puppies are found immunization is carried out at earliest possible date. As an important preventive measure. pre-exposure vaccination is strongly recommended for anyone who is at continuous, frequent or increased risk for exposure to rabies virus [18]. It is recommended that laboratory staff, veterinarians, and anyone who works with animals and wildlife receive pre-exposure prophylaxis to reduce their occupational risk of infection [18].

Also sustaining control demands political and financial backing to maintain the anti-rabies campaign as well as the logistic and human resource capacity to deliver vaccine, and knowledge of, and access to, target populations. On-going collection of data through surveillance system to monitor and evaluate the economic and technical efficiency of campaigns is necessary to ensure objectives are being achieved, and surveillance must be continuous following eradication to detect re-emergence of the virus promptly [19]. Acoording to *Neil* [20] lack of reporting on rabies data by most developing countries is disconcerting. Examination of data on WHO and OIE web sites showed that information was frequently missing for an entire year or more in several countries. WHOP focuses mainly on human disease, and will most likely receive data from medical health authorities. In contrast, the animal disease focus of OIE suggests that vetenary services will submit rabies data to this body [20]. For a long duration, Sri Lanka has very effective rabies surveillance and reporting system. Epidemiology unit of Ministry of Health, Sri Lanka has official publication; Epidemiological Bulletin which updates the situation of important diseases including rabies [5–7, 10–14]. Sri Lanka has achieved tremendous success in controlling and eradicating human rabies over the last few decades and those strategies would be a good model for countries with high prevalence of human rabies in planning their strategies in controlling rabies.

## Conclusion

We audited burden of animal exposure and post exposure therapy in a tertiary care hospital In Sri Lanka for 5 years. Numbers of animal exposures and cost incurred for PET is colossal. While dogs were responsible for most of the bites, there are number of other animals bites used to present for PET. Even though, most of the offending dogs remained healthy, there should not be room for complacency in PET. In spite of combined efforts of local and state health departments, animal control officers and the vetenary community, rabies is an important community health problem in Sri Lanka. However, more understanding is needed about PET to make more efficient and tailor made to conditions in Sri Lanka. Furthermore, public should be made aware of prevention of animal bite particularly among vulnerable groups. Continued surveillance and rabies control is still necessary to protect public health and to reduce the need of PET. Another way to reduce the need for rabies PET is to improve vaccination rate of both domestic pets and stray animals. Other methods such as use of contraception for the animals to reduce their population would of use. By educating school children and general public about responsible pet ownership, bite prevention, precautions in dealing with wildlife, and appropriate wound care may also help to reduce need of PET.

## Ethical clearance

Written permission was obtained from the authority, Teaching Hospital, Peradeniya for data collection and publication. As the study was on hospital records which did not disclose the identity of the patients, Ethical review committee of Faculty of Medicine, University of Peradeniya stated that hospital administrative permission alone will be adequate to carry out the study.

## Abbreviations

FAT: Fluorescent Antibody Test
MRI: Medical Research Institute
PET: post exposure treatment
THP: Teaching Hospital Peradeniya
WHO: World Health Organization

## References

[1] WHO rabies fact sheet No 99;September 2014.

[2] Fevre EM, Kaboyo RW, Persson V, Edelsten M, Coleman PG, Cleaveland S (2005). The epidemiology of animal bite injuries in Uganda and projections of the burden of rabies. Trop Med Int Health.

[3] Karunanayake D, Matsumoto T, Wimalaratne O, Nanayakkara S, Perera D, Nishizono A (2014). Twelve years of rabies surveillance in Sri Lanka,1999-2010. PLoS Negl Trop Dis.

[4] Hasler B, Hiby E, Gilbert W, Obeyesekere N, Bennani H, Rushton J (2014). A one health framework for the evaluation of rabies control programmes:A case study from Colombo city,Sri Lanka. PLoS Negl Trop Dis.

[5] Rabies(Part 1).WER Sri Lanka September 2014;41(38):1-4.

[6] Rabies(Part 11).WER Sri Lanka September 2014;41(39):1-4.

[7] Rabies.WER Sri Lanka May 2011;38(19):1-2.

[8] Smith JS (1996). New aspects of rabies with emphasis on epidemiology, diagnosis and prevention of the disease in the United States. Clin Microbiol Rev.

[9] WHO Expert Consultation on Rabies ;Second Report:WHO Technical report series 982. 24069724

[10] Epidemiology bulletin Sri Lanka First,Second,Third and Fourth Quarters 2007. http://www.epid.gov.lk

[11] Epidemiology bulletin Sri Lanka First,Second,Third and Fourth Quarters 2009. http://www.epid.gov.lk

[12] Epidemiology bulletin Sri Lanka First,second,Third and Fourt Quarters 2010. http://www.epid.gov.lk

[13] Epidemiology bulletin Sri Lanka First,Second,Third and Fourth Quarters 2011. http://www.epid.gov.lk

[14] Epidemiology bulletin Sri Lanka First,Second,Third and Fourth Quarters 2012. http://www.epid.gov.lk

[15] Salve H, Kumar S, Rizwan SA, Raj SK, Kant S, Panday CS. Feasibility of sustainable provision of intradermal post exposure prophylaxis against rabies at primary care level-evidence from rural Haryana. BMC Health Service Research 2014;278:1-6 10.1186/1472-6963-14-278 PMC4076435 24965875

[16] Shah V, Bala DV, Thakker J, Dalal A, Shah U, Chauhan S, Govani K. Epidemiological determinants of animal bite cases attending the anti-rabies clinic at V S General Hospital, Ahmedabad. Healthlin.e 2012;3(1):66-8

[17] Moore DA, Sischo WM, Hunter A, Miles T. Animal bite epidemiology and surveillance for rabies postexposure prophylaxis. JAVMA. 2000;217(2):190-4 10.2460/javma.2000.217.190 10909457

[18] Crowcroft NS, Thampi N (2015). The prevention and management of rabies:Clinical review. BMJ.

[19] Gondal G, Wright AE (2011). Human rabies in the WHO southeast Asia Region: Forward Steps for Elimination: Review article. Advances in Preventive Medicine.

[20] Nel LH. Discrepancies in datareporting for rabies, Africa. Emerging infectious diseases. 2013;12(4):529-33 10.3201/eid1904.120185 PMC3647406 23628197

